# Radiation Exposure Decreases the Quantity and Quality of Cardiac Stem Cells in Mice

**DOI:** 10.1371/journal.pone.0152179

**Published:** 2016-05-19

**Authors:** Lan Luo, Yoshishige Urata, Chen Yan, Al Shaimaa Hasan, Shinji Goto, Chang-Ying Guo, Fang-Fang Tou, Yucai Xie, Tao-Sheng Li

**Affiliations:** 1 Department of Stem Cell Biology, Nagasaki University Graduate School of Biomedical Sciences, 1-12-4 Sakamoto, Nagasaki, 852-8523, Japan; 2 Jiangxi Cancer Hospital, Nanchang, Jiangxi, 330029, PR China; 3 Department of Cardiology, Shanghai Ruijin Hospital, Shanghai Jiao Tong University School of Medicine, Shanghai, China; Georgia Regents University, UNITED STATES

## Abstract

Radiation exposure may increase cardiovascular disease risks; however, the precise molecular/cellular mechanisms remain unclear. In the present study, we examined the hypothesis that radiation impairs cardiac stem cells (CSCs), thereby contributing to future cardiovascular disease risks. Adult C57BL/6 mice were exposed to 3 Gy γ-rays, and heart tissues were collected 24 hours later for further experiments. Although c-kit-positive cells were rarely found, radiation exposure significantly induced apoptosis and DNA damage in the cells of the heart. The *ex vivo* expansion of CSCs from freshly harvested atrial tissues showed a significantly lower production of CSCs in irradiated mice compared with healthy mice. The proliferative activity of CSCs evaluated by Ki-67 expression was not significantly different between the groups. However, compared to the healthy control, CSCs expanded from irradiated mice showed significantly lower telomerase activity, more 53BP1 foci in the nuclei, lower expression of c-kit and higher expression of CD90. Furthermore, CSCs expanded from irradiated mice had significantly poorer potency in the production of insulin-like growth factor-1. Our data suggest that radiation exposure significantly decreases the quantity and quality of CSCs, which may serve as sensitive bio-parameters for predicting future cardiovascular disease risks.

## Introduction

The heart is generally considered to be a radioresistant organ due to its composition of postmitotic cardiac myocytes and the extremely low proliferative activity of its endothelial cells and connective tissue cells. However, a cohort study among atomic bomb survivors in Hiroshima and Nagasaki indicated that radiation exposure over 0.5 Gy significantly elevated the risks of both stroke and heart disease [1]. Increased risks of ischemic heart disease are also found in nuclear workers from the Mayak nuclear facility in the Russian Federation, site of the Kyshtym disaster, and in breast cancer patients who have received radiotherapy [2,3]. Experimental studies in ApoE(-/-) mice have further demonstrated that ionizing radiation accelerates atherosclerosis of the coronary arteries by inducing inflammation and damaging the microvascular and endocardial cells [4,5]. However, the molecular and cellular mechanisms of radiation-induced cardiovascular disease risks have not yet been fully elucidated because of the lack of experimental models/approaches for evaluation of these mechanisms [6–8].

The resident tissue-specific stem/progenitor cells, a rare population of cells that are able to renew themselves and differentiate into other cell types, are known to play crucial roles in tissue/organ repair and regeneration in response to physiological turnover or pathological damage throughout life. Growing evidence has also shown that the injured tissue-specific stem/progenitor cells mainly contribute to carcinogenesis, one of the major problems at the late phase after radiation [9,10]. Our recent studies have further confirmed a very high radiosensitivity of hematopoietic stem cells and muscle stem cells [11,12]. Therefore, it is quite possible that the changes in the quantity and quality of tissue-specific stem/progenitor cells will serve as indirect indicators of cancer and non-cancer risks. Indeed, many radiobiologists have started to evaluate the radiation-induced health problems by focusing on the tissue-specific stem/progenitor cells [13,14]. The recent success in the identification and *ex vivo* expansion of cardiac stem cells (CSCs) from heart tissues of animals and human beings has provided us a new feasible approach to evaluate radiation-induced cardiovascular disease risks.

Here, by exposing healthy adult mice to 3 Gy γ-rays, we evaluated how whole-body radiation exposure impairs CSCs. We are also interested in finding reliable and sensitive bio-parameters regarding the radiation-induced changes in the quantity and quality of CSCs that are useful for predicting future potential cardiovascular disease risks.

## Materials and Methods

### Animals

Adult (10-12-week-old) male C57BL/6 mice (CLEA Japan, Inc.) were used for the experiments. This study was approved by the Institutional Animal Care and Use Committee of Nagasaki University (No. 1108120943–8), and all animal procedures were performed in accordance with the institutional and national guidelines.

### Radiation exposure

Whole-body radiation exposure was performed by exposing the mice to 3 Gy γ-rays with a Cs source at a dose rate of 0.886 Gy/min with a PS-3100SB γ-ray irradiation system (Pony Industry Co., Ltd. Osaka, Japan) [15]. Age-matched healthy mice were used as controls. The mice were euthanized by severing the aorta under general anesthesia with intraperitoneal injection of 160 mg/kg pentobarbital 24 hours after irradiation, and the hearts were quickly injected with 5 ml cold cardioplegic solution (Mochida Pharmaceutical Co., LTD.). The ventricular tissues were embedded in OCT compound for histological analysis, and the atrial tissues were collected for *ex vivo* expansion of CSCs as described below.

### Histological analyses

The ventricular tissues were sectioned in 7-μm sections and fixed with 4% paraformaldehyde. Apoptosis in the heart tissues was detected using a TACS 2 TdT-Fluor In Situ Apoptosis Detection Kit (Trevigen, Inc.) according to the manufacturer’s instructions. The cell nuclei were stained with 4’, 6-diamidino-2-phenylin-dole (DAPI), and terminal deoxynucleotidyl transferase dUTP nick-end labeling (TUNEL)-positive apoptotic cells were counted under a fluorescent microscope with 200-fold magnification. Fifteen fields per section were randomly selected for quantitative counting.

The DNA damage in cardiomyocytes and c-kit-positive stem cells was also identified by immunostaining with rabbit polyclonal anti-mouse 53BP1 antibody (Abcam), followed by double staining with mouse monoclonal anti alpha-sarcomeric actin antibody (Sigma-Aldrich) or goat polyclonal anti-mouse c-kit antibody (R&D Systems). Appropriate secondary antibodies conjugated with Alexa fluorochromes were used, and nuclei were then stained with DAPI. The positively stained cells were counted under a fluorescent microscope as described above.

### *Ex vivo* expansion of CSCs

Mouse CSCs were expanded using methods similar to those previously described [16]. Briefly, atrial tissues from mice were minced into small fragments and cultured as “explants” on 6-cm culture dishes coated with 15 μg/ml fibronectin (CORNING). Within 1 week, stromal-like flat cells and phase-bright round cells emerged from the tissue fragments, and they became confluent at approximately 2 weeks. The outgrowth of CSCs was harvested using 0.25% trypsin (Gibco) at 2 weeks, counted using a Nucleo Counter cell-counting device (Chemotetec A/S, Denmark), and then passaged for cell expansion. Twice-passaged CSCs were used for the following experiments as indicated. All cells were cultured in a 5% CO_2_ incubator at 37°C using IMDM basic medium (Gibco) supplemented with 10% fetal bovine serum (HyClone), 100 units/ml penicillin G and 10 μg/ml streptomycin (WAKO, Japan).

### Characterization of CSCs

The expression levels of c-kit and CD90 in CSCs were estimated by immunostaining [17]. In brief, twice-passaged CSCs (1x10^4^/well) were cultured in 8-well chamber culture slides (Lab-Tek, Thermo Scientific Nunc) coated with 15 μg/ml fibronectin (CORNING). The cells were fixed in 4% paraformaldehyde for 10 min after 3 days of culture. After blocking, the cells were incubated with rat anti-mouse c-kit conjugated with PE antibody (eBioscience) or rat monoclonal anti-mouse CD90 antibody (Abcam), followed by donkey anti-rat Alexa Flour 488-conjugated secondary antibody. Nuclei were stained with DAPI, and the positively stained cells were counted under a fluorescent microscope with 200-fold magnification. Twenty fields per section were randomly selected for quantitative counting.

### Evaluation of cell proliferation, senescence, and DNA damage

To evaluate the cell proliferation, senescence [18], and DNA damage [11], twice-passaged CSCs (1x10^4^/well) were seeded on 8-well chamber culture slides as mentioned above. After 3 days of culture, the cells were fixed in 4% paraformaldehyde for 10 min. After blocking, the cells were incubated with rat monoclonal anti-mouse Ki-67 antibody (Dako), rabbit polyclonal anti-mouse telomerase reverse transcriptase-C-terminal antibody (Abcam), or rabbit polyclonal anti-mouse 53BP1 antibody (Abcam). Appropriate second antibodies conjugated with Alexa fluorochromes were used. The nuclei were stained with DAPI, and the positively stained cells were counted under a fluorescent microscope with 200-fold magnification. Twenty fields per section were randomly selected for quantitative counting.

### ELISA

Conditioned media was collected from twice-passaged CSCs 3 days after culture in 8-well chamber culture slides. A mouse VEGF ELISA kit and a mouse/rat IGF-1 ELISA kit (R&D Systems) were used to measure the levels of VEGF and IGF-1 in the conditioned medium [18].

### Statistical analysis

All of the results are presented as the mean ± SEM. The statistical significance was determined by Student’s *t*-test (EXCEL). Differences were considered significant when *P*<0.05.

## Results

### Whole-body exposure to 3 Gy γ-rays induced apoptosis and DNA damage in the heart tissues of mice

**Fig 1:**
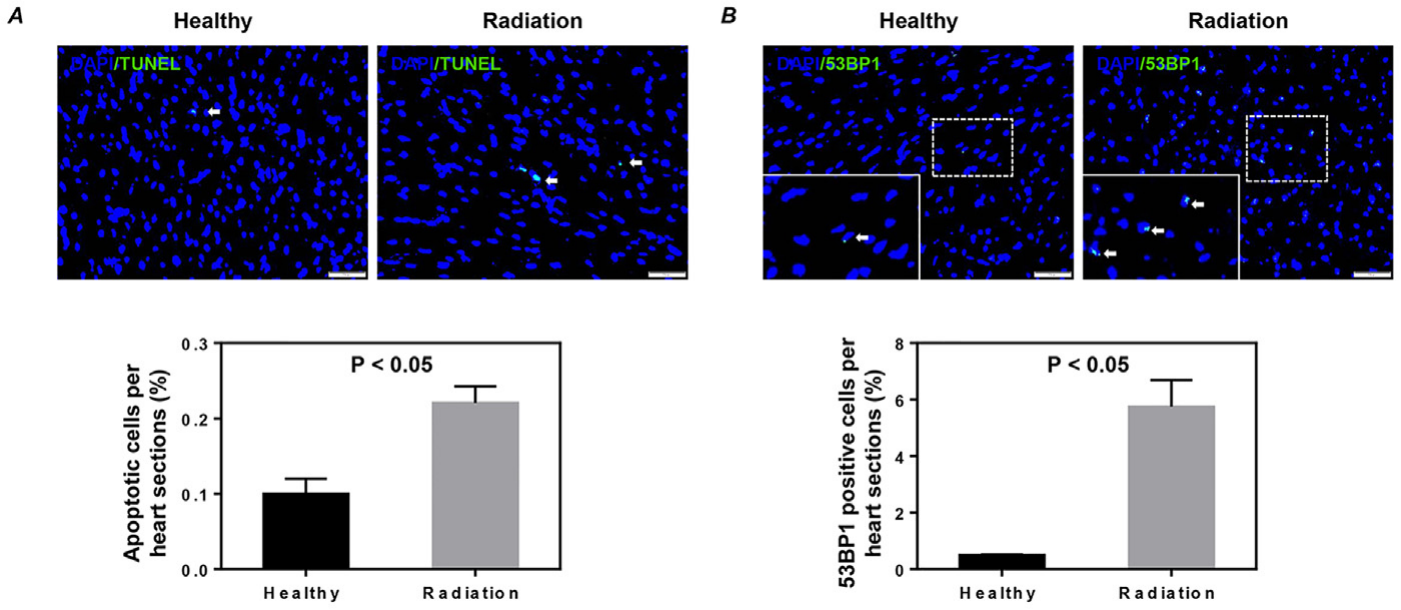
Apoptosis and DNA damage in the heart after whole-body radiation exposure. Apoptosis (A) and DNA damage (B) were detected by TUNEL assay and immunostaining with 53BP1, respectively. Representative images (upper) and quantitative data (lower) are shown. Arrows in (A) and (B) indicate positively stained cells. The nuclei were stained with DAPI. Scale bar: 50 μm, 20 μm for magnification. Values shown are the mean ± SEM from 3 independent experiments.

First, we investigated apoptosis and DNA damage by immunohistological analysis. We found that whole-body exposure of adult mice to 3 Gy γ-rays significantly induced apoptosis and DNA damage in the heart sections (*P*<0.05, Fig 1).

**Fig 2:**
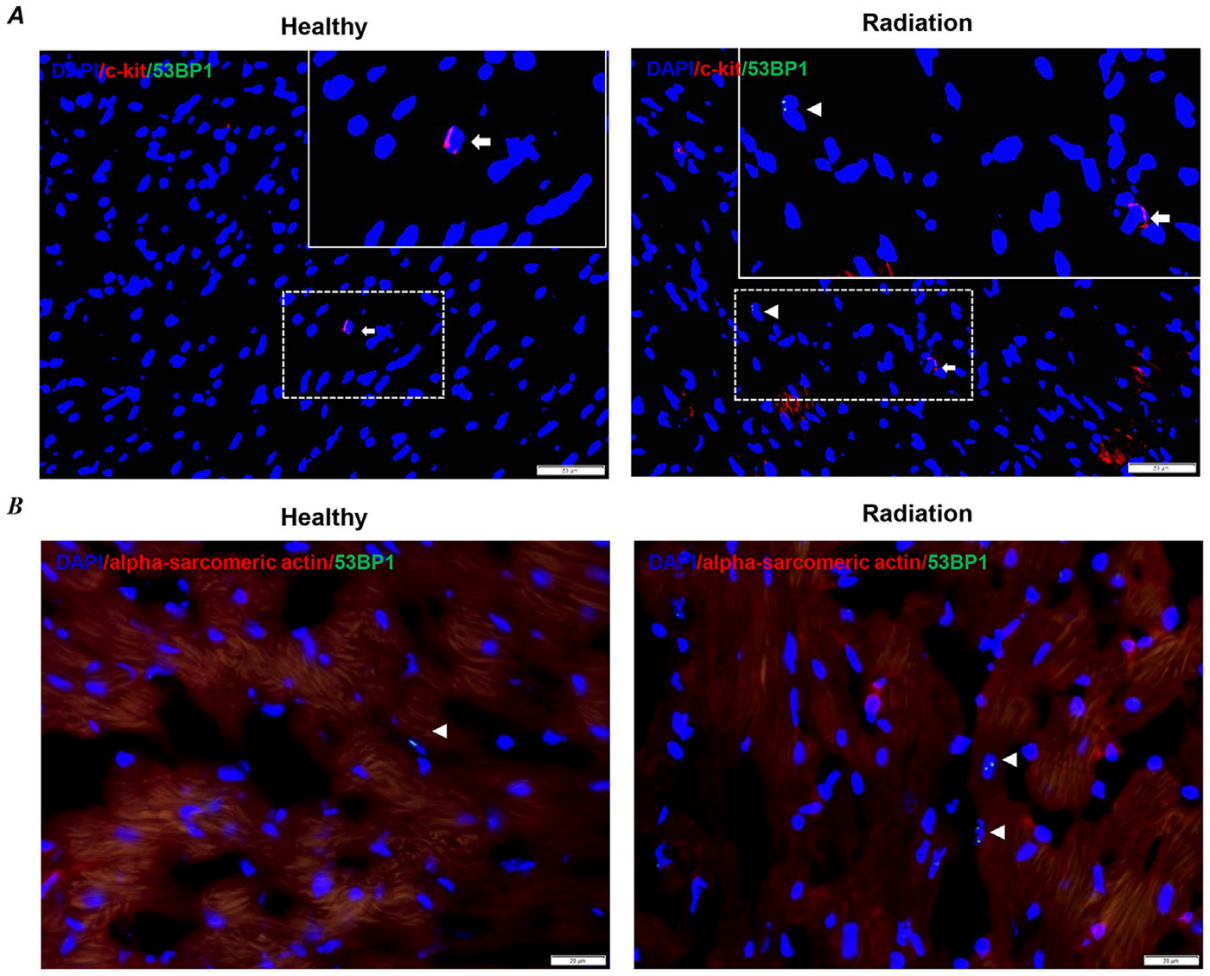
DNA damage in c-kit-positive stem cells and cardiomyocytes on heart sections. (A) Representative images of DNA damage (53BP1 foci) in c-kit-positive stem cells. Arrows and arrowheads indicate the c-kit-positive stem cells (red, c-kit) and 53BP1 foci (green dots), respectively. The nuclei were stained with DAPI. Scale bars: 50 μm and 10 μm (left), 20 μm (right) for magnification. (B) Representative images of DNA damage (53BP1 foci) in cardiomyocytes. Arrowheads indicate the 53BP1 foci (green dots) in cardiomyocytes (red, alpha sarcomeric actin), respectively. The nuclei were stained with DAPI. Scale bars: 20 μm.

Double staining was further performed to confirm the localization of DNA damage, which occurred in the c-kit-positive stem cells, cardiomyocytes, or other tissue cells. The c-kit-positive stem cells were rarely found within the heart tissues (Fig 2A), and it was difficult for us to quantify the DNA damage in these c-kit-positive stem cells. Only a few of the cardiomyocytes were positively stained with 53BP1 (Fig 2B), indicating the radioresistance of the cardiomyocytes. Therefore, we thought that whole-body radiation exposure may induce DNA damage in cells other than the c-kit-positive stem cells and cardiomyocytes within the heart tissues.

### Whole-body exposure to 3 Gy γ-rays significantly decreased the outgrowth of CSCs and altered their phenotypes

**Fig 3:**
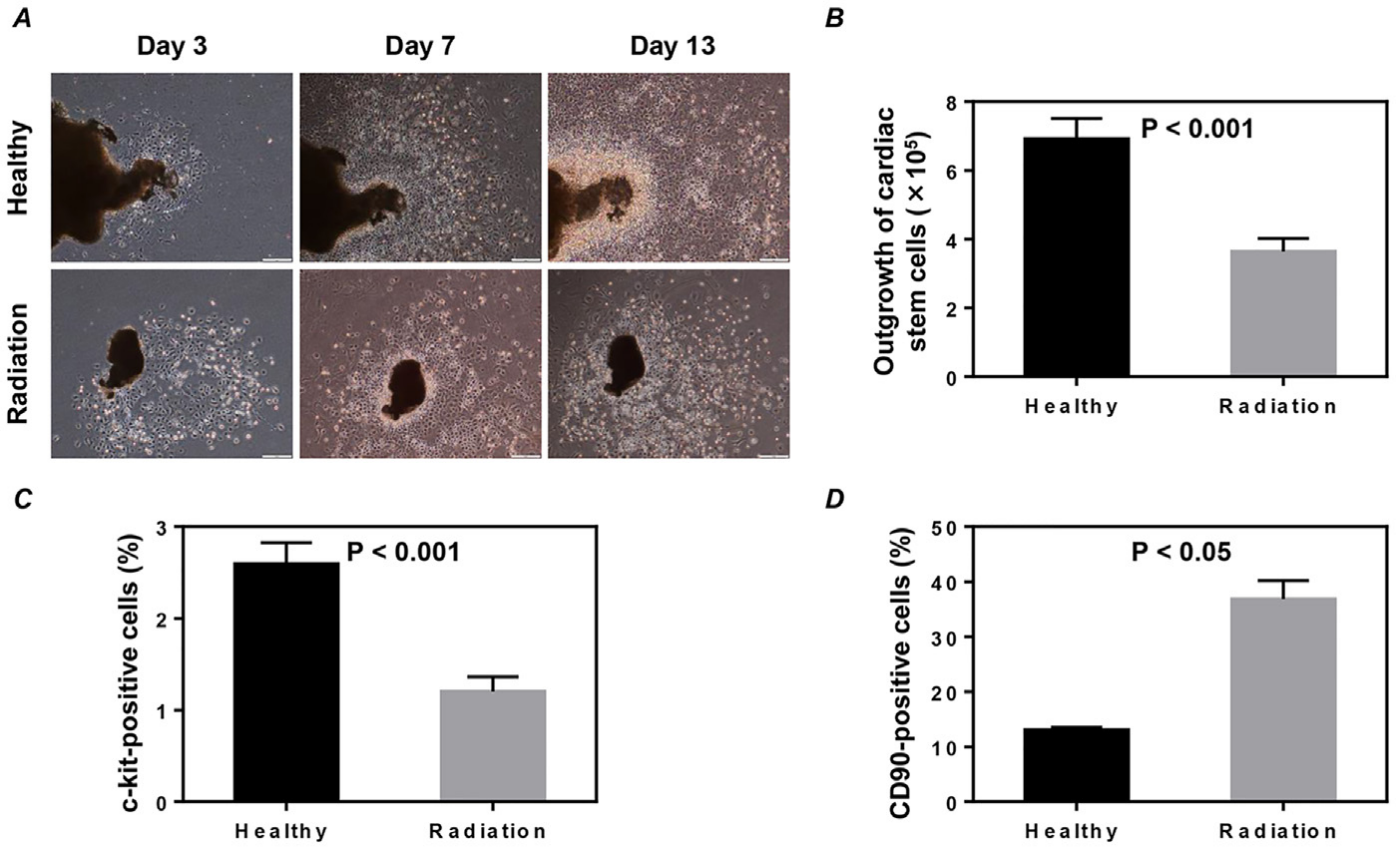
*Ex vivo* expansion and phenotypic characterization of cardiac stem cells (CSCs). (A) Representative images of CSCs expanded from the explants of heart atrial tissues. Scale bar: 50 μm. (B) CSCs were harvested at day 14, and the number of total collected CSCs from each mouse was directly counted. Values shown are the mean ± SEM of 6 independent experiments. The phenotypic characterization of c-kit (C) and CD90 (D) was determined by immunostaining of twice-passaged CSCs. Quantitative data of the expression of c-kit and CD90 were obtained by counting positively stained cells from 20 randomly selected fields. Values shown are the mean ± SEM from 6 independent experiments in C and 3 independent experiments in D.

We then tried to perform *ex vivo* expansion of CSCs, a cardiac type of mesenchymal stem cells, using a defined protocol [16]. At day 3 after the initiation of culture, a layer of fibroblast-like cells expanded from plants over which small, phase-bright cells migrated out from the “explants”. Although the cell shape appeared similar between the groups (Fig 3A), a significantly lower number of CSCs were harvested from the heart atrial tissues of irradiated mice than from the heart atrial tissues of healthy mice two weeks after culture (*P*<0.001, Fig 3B).

CSCs expanded from atrial tissues had been shown to be a mixed population [19]. To examine whether radiation exposure would also change the phenotypic characterization of CSCs, we stained the twice-passaged CSCs with a common stem cell marker of c-kit and a mesenchymal marker of CD90. Compared to these CSCs expanded from the atrial tissues of healthy mice, CSCs from the atrial tissues of irradiated mice expressed significantly lower c-kit (*P*<0.001, Fig 3C) but higher CD90 (*P*<0.05, Fig 3D).

### Whole-body exposure to 3 Gy γ-rays did not affect proliferation but significantly induced cell senescence and DNA damage of CSCs

**Fig 4:**
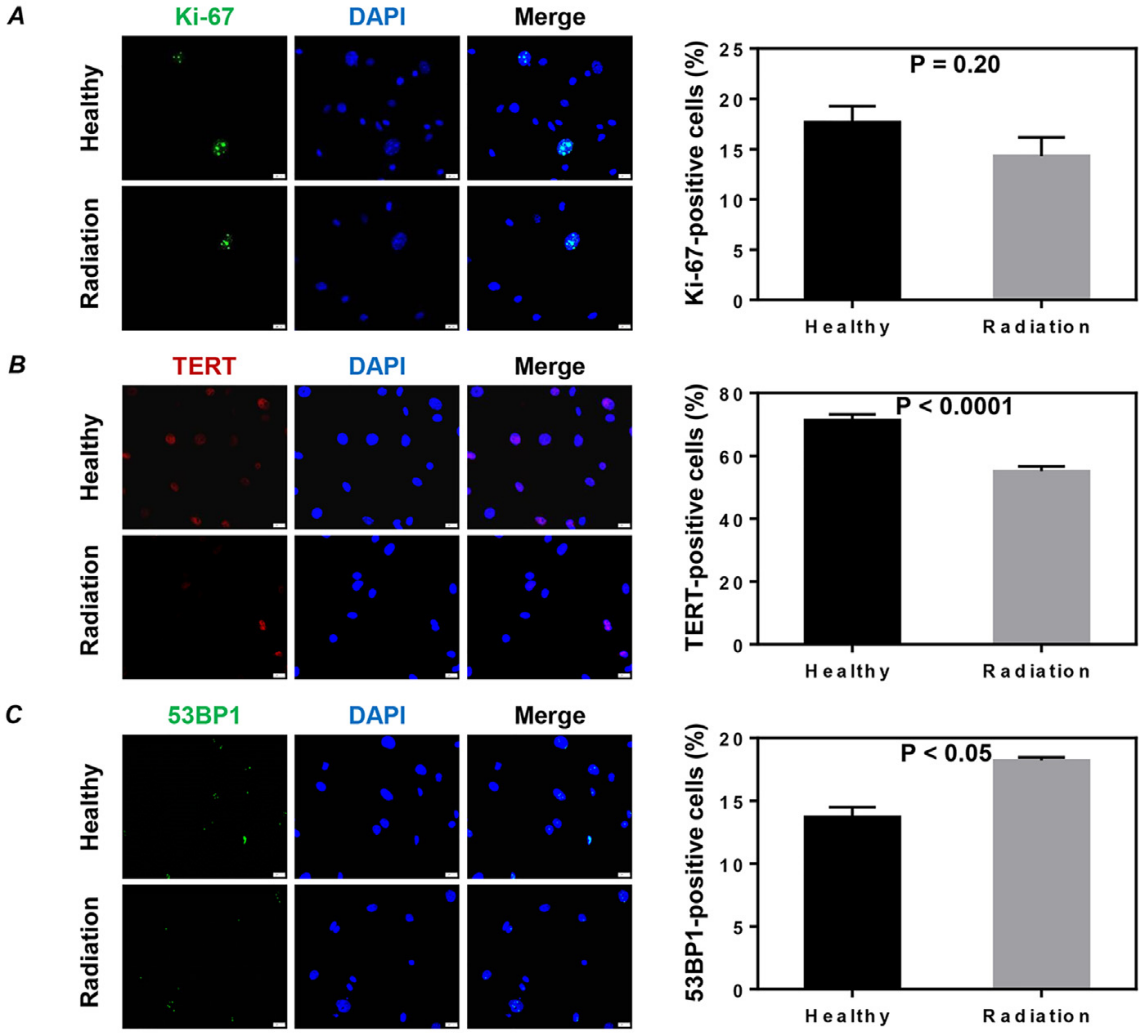
Proliferation, telomerase activity and DNA damage of cardiac stem cells (CSCs). The proliferation (A), telomerase activity (B) and DNA damage (C) of CSCs were evaluated by immunostaining with Ki-67, telomerase reverse transcriptase (TERT), and 53BP1, respectively. Representative images (left) and quantitative data (right) are shown. The nuclei were stained with DAPI. Scale bar: 20 μm. Values shown are the mean ± SEM from 6 independent experiments in A and B and 3 independent experiments in C.

The proliferative activity of CSCs was evaluated by the expression of Ki-67. We found that the expression of Ki-67 was not significantly different between the two groups (*P* = 0.20, Fig 4A). The cell senescence of CSCs was evaluated by immunostaining with telomerase reverse transcriptase (TERT). The expression of TERT was significantly lower in the CSCs of irradiated mice than in those of healthy mice (*P*<0.0001, Fig 4B). The percentage of cells with 53BP1 foci, a popular DNA damage marker, was also observed to be higher in the CSCs of irradiated mice than in those of healthy mice (*P*<0.05, Fig 4C).

### Whole-body exposure to 3 Gy γ-rays impaired the ability of CSCs to produce IGF-1

**Fig 5:**
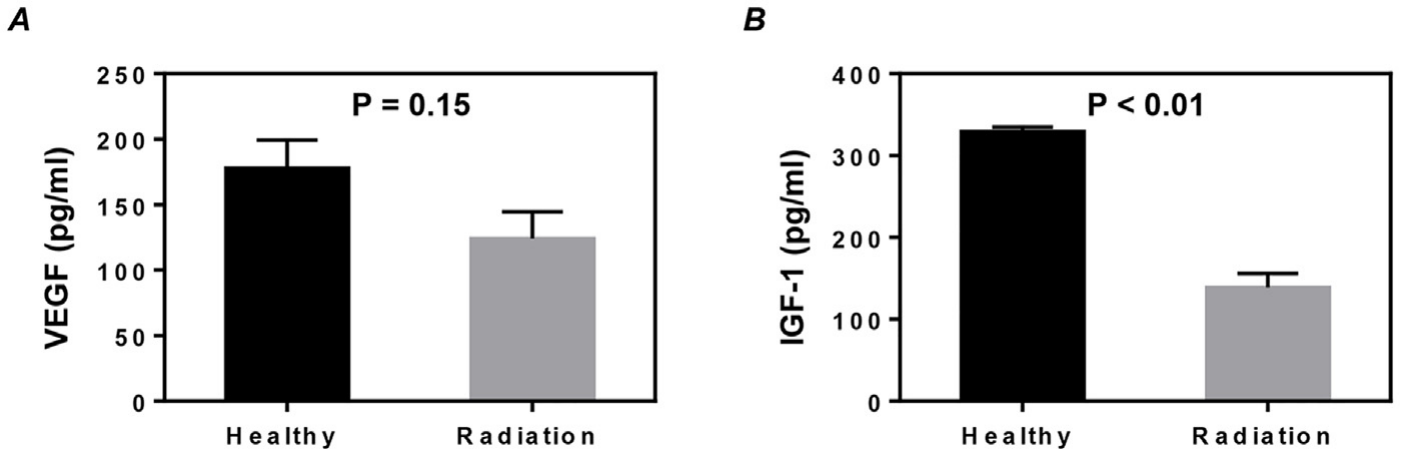
Growth factor production of cardiac stem cells (CSCs). The supernatants of twice-passaged CSCs were collected to measure the concentration of vascular endothelial growth factor (VEGF) (A) and insulin-like growth factor-1 (IGF-1) (B) by ELISA. Values shown are the mean ± SEM from 3 independent experiments.

The implantation of stem cells for myocardial repair was generally thought to occur mainly through the paracrine mechanism rather than by direct regeneration [20]. Therefore, we also examined whether radiation exposure would impair the production of vascular endothelial growth factor (VEGF) and insulin-like growth factor-1 (IGF-1), two important beneficial factors for myocardial repair released by CSCs. Compared with the CSCs expanded from the hearts of healthy mice, CSCs from the irradiated mice showed a slight decrease in the production of VEGF (*P* = 0.15, Fig 5A) and a significant decrease in the production of IGF-1 (*P*<0.01, Fig 5B).

## Discussion

Stem cells are known to play critical roles in tissue homeostasis. Ionizing radiation exposure can disrupt the tissue homeostasis through the depletion and accumulation of genetic abnormalities in these radiosensitive tissue-specific stem/progenitor cells, which will finally result in the loss of tissue function. By exposing the adult mice to 3 Gy γ-rays, we evaluated the radiation-induced injury to cardiac stem cells in terms of quantity and quality using various experimental techniques.

We initially investigated apoptosis and DNA damage of cells in the heart tissues after whole-body radiation exposure by immunostaining. Although radiation significantly induces apoptosis and DNA damage, a few of the cardiomyocytes were involved. This confirmed the radioresistance of cardiomyocytes. We then suspected that the cells within the heart tissues that underwent apoptosis and exhibited DNA damage might be the radiosensitive stem/progenitor cells. As there was no consensus on the marker(s) for the identification of CSCs, we used a common stem cell marker of c-kit. Unfortunately, the c-kit positive stem cells were very rarely found in the heart tissues.

In the past decade, *ex vivo* expansion of the very small population of resident stem/progenitor cells from the heart tissues of human, rat or mouse have been well established [17,19]. To further examine whether whole-body radiation exposure impaired the radiosensitive stem/progenitor cells in the heart, we expanded stem cells from the heart tissues of mice using a method developed by our group [16]. Despite the well-trained skills, fewer stem cells were outgrown from the atrial tissues of the mice that received whole-body exposure to 3 Gy γ-rays compared with that of the healthy controls. Strangely, telomerase activity was significantly decreased in twice-passaged CSCs from the hearts of irradiated mice, but the proliferative potency was only slightly impaired. This might be due to the high sensitivity of telomeres to ionizing irradiation [21]. Additionally, we observed an increase in the formation of 53BP1 foci in the nuclei of twice-passaged CSCs from the irradiated mice. These results clearly suggested that whole-body exposure of adult mice to 3 Gy γ-rays significantly damaged the cardiac stem cells.

The present study was designed to find sensitive and reliable bio-parameter(s) of the damage to stem cells, which might be used for predicting future radiation-induced cardiovascular risks. Previous studies have suggested that the small fraction of c-kit-positive cells are cardiac stem cells [22,23], but the role of c-kit-positive cells in heart regeneration remains controversial [24]. It has recently been found that the c-kit^-^/CD90^-^ cells also played an indispensable role in the regenerative potential of CSCs [25]. Otherwise, CSCs likely serve to repair an injured heart through direct and indirect (paracrine) mechanisms, and growing evidence suggests that the paracrine mechanism mediated by factors secreted by CSCs, such as VEGF and IGF-1, plays an essential role in myocardial repair [20,26]. VEGF is well-known to promote angiogenesis and induce endothelial cell proliferation and tube formation. IGF-1 is one of the most important factors for inhibiting apoptosis and stimulating the growth and proliferation of stem cells. Therefore, we examined whether radiation exposure altered the phenotypic characterization of CSCs or their potency in releasing paracrine factors. Our results showed that the percentage of c-kit-positive cells was significantly decreased, while the percentage of CD90-positive-cells was significantly increased in the twice-passaged CSCs from the irradiated mice. Furthermore, CSCs from the irradiated mice showed a slight decrease in VEGF production and a significant decrease in IGF-1 production. The poor release of paracrine factors suggested a functional impairment of CSCs. To the best of our knowledge, this is the first systemic experimental examinations of radiation-induced damage to CSCs. We only selected 3 Gy γ-rays to find the potential bio-parameter(s) for detecting the radiation-induced injuries in CSCs. Our data suggested that whole-body exposure of adult mice to 3Gy γ-rays significantly decreased the quantity and quality of CSCs. Many bio-parameters selected in this study, including cell outgrowth, cell phenotypes, DNA damage, telomerase activity and growth factor production could sensitively detect the damage to CSCs. However, many other important parameters of the quality of CSCs, such as cell differentiation, were not examined because our study was primarily designed to determine reliable and sensitive markers for evaluating radiation-induced damage to CSCs. However, it remained controversial and there were technical challenges regarding the myocardial differentiation of CSCs. Further experiments with a wide dose range of irradiation, especially with lower dosages, are required to confirm the most sensitive and reliable bio-parameter(s) for detecting radiation-induced damage of CSCs.

CSCs are known to participate in heart regeneration due to physiological turnover or pathological damage. The decrease in the quantity and quality of CSCs will result in insufficient regeneration/repair to the heart, which thereby contributes to cardiovascular diseases. Theoretically, the changes in the quantity and quality of CSCs soon after radiation exposure may be useful for predicting the long-term future cardiovascular disease risks. However, beyond the CSCs, the damage to other cells, including the endothelial progenitor cells, are also known to be associated with cardiovascular disease risks [27, 28]. Therefore, more studies, including an *in vivo* experiment, will be required to confirm whether the decreased quantity and quality of CSCs will really be associated with impaired regenerative potency and with increased cardiovascular disease risks.

In summary, many bio-parameters from the present study indicated that whole-body exposure of adult mice to 3 Gy γ-rays significantly decreased the quantity and quality of CSCs. The data from the present study provided novel information for selecting reliable bio-parameters of radiation-induced injuries of CSCs. As CSCs appear to be highly sensitive to radiation exposure, the damage to CSCs may finally help us to predict future cardiovascular disease risks after radiation, especially in response to low dose exposures.
